# Co-overexpression of RIOK1 and AKT1 as a prognostic risk factor in glioma

**DOI:** 10.7150/jca.60596

**Published:** 2021-07-25

**Authors:** Yiwei Wang, Xiaochen Xie, Shu Li, Dongyong Zhang, Heyu Zheng, Min Zhang, Zhong Zhang

**Affiliations:** 1 Department of Human Anatomy, Shenyang Medical College, Shenyang City, Liaoning Province 110034, P.R. China.; 2 Department of Pathology, College of Basic Medical Sciences, Shenyang Medical College, Shenyang City, Liaoning Province 110034, P.R. China.; 3 Department of Endocrinology and Metabolism, Institute of Endocrinology, Liaoning Provincial Key Laboratory of Endocrine Diseases, The First Affiliated Hospital of China Medical University, China Medical University, Shenyang, Liaoning, 110001, P.R. China.; 4 Department of Neurosurgery, First Affiliated Hospital of China Medical University, Heping District, Shenyang City, Liaoning Province, 110001, P.R. China

**Keywords:** RIOK1, AKT1, c-Myc, GBM

## Abstract

Glioblastoma multiforme (GBM) is one of the most frequent primary malignancies of the brain. Although the treatment strategy has significantly improved, patient prognosis remains poor. *In vitro* studies have shown that the right open reading frame kinase 1/protein kinase B (RIOK1-AKT) signaling pathway plays an important role in the malignant phenotype of glioma cells. This study aimed to investigate the co-expression of RIOK1 and ATK in glioma tissues and its clinical significance. Compared with normal tissues, RIOK1 and AKT1 expression were significantly upregulated in glioma tissues. In addition, patients with higher World Health Organization staging grades had increased RIOK1 and AKT1 expression levels, and RIOK1 and AKT1 expression were positively correlated. Notably, both RIOK1 and AKT1 expressions were correlated with poor prognosis. *In vitro* experiments showed that silencing RIOK1 inhibited the proliferation, migration, and invasion of glioma cell lines by suppressing AKT and c-Myc expression. These results indicate that the RIOK1-AKT1 axis could play an important role in GBM progression.

## Introduction

Glioblastoma multiforme (GBM) is one of the most frequent primary malignancies of the brain, accounting for 60% of primary intracranial tumors and 80% of the primary intracranial malignancies 1, 2. Despite advances in surgery, radiotherapy and chemotherapy technology, patient prognosis remains poor, especially for those with high-grade disease 3-6. It is therefore important to explore potential molecular mechanisms that promote the malignant progression of GBM, which might identify new treatment strategies.

The right open reading frame kinase (Rio kinase) family consists of three members (RIOK1, RIOK2 and RIOK3) that are involved in ribosome synthesis and processing *in vivo* 7, 8. RIOK2 expression is increased in non-small cell carcinomas such as lung squamous cell carcinoma and adenocarcinoma and is associated with the degree of differentiation and pathological stage 9, 10. RIOK3 can also promote the proliferation, invasion and migration of glioma cells through protein kinase B/mammalian target of rapamycin (AKT/mTOR) signaling pathway 11. RIOK1 is an important member of the atypical serine/threonine kinase family. A previous study demonstrated that RIOK1 is highly expressed in colorectal cancer cells and promotes proliferation and invasion by activating the nuclear factor-κB signaling pathway of Ras mutant tumor cells, but it has no effect on Ras wild-type cells 10. In addition, silencing RIOK1 suppresses the proliferation, migration, and invasion of breast, lung and colon cancer cell liens 9. RIOK1 and RIOK2 are also highly expressed in glioma cell lines; overexpressing RIOK1/2 contributes to proliferation, while their knockdown induces apoptosis and renders cells more sensitive to chemotherapy 12. AKT1 is an important part of the AKT signaling pathway, and its upregulation is associated with poor prognosis in patients with GBM 13. In a number of tumor types, increased AKT1 activity can promote tumorigenesis and development 14-16. RIOK1 plays important roles in many kinds of tumors, but its expression in different types of glioma cells and its effect on biological behavior are still unclear. It is therefore essential to investigate RIOK1 and AKT1 expression in glioma to identify new therapeutic approaches and prognostic biomarkers.

In this study, we confirmed that RIOK1 and AKT1 levels significantly increased in GBM tissues and were positively associated with tumor malignancy, while knockdown of RIOK1 inhibits glioma cell growth through AKT1 and c-Myc *in vitro*. Our results highlight RIOK1 as a novel therapeutic target in GBM.

## Materials and Methods

### Gene expression analysis

RIOK1 and AKT1 mRNA expression data were obtained from The Cancer Genome Atlas (TCGA) GBMLGG (689 gliomas) and GTEX (1157 normal tissues) datasets. We obtained the scatter diagram of RIOK1 and AKT1 expression correlation from TCGA GBMLGG (https://portal.gdc.cancer.gov/). RNAseq data was given in TPM (transcripts per million reads) format, and expression levels compared between samples after log2 transformation.

### Patients and tissue specimens

A total of 106 glioma tissue samples were collected by the Department of Neurosurgery of the First Affiliated Hospital of China Medical University from 2012 to 2018. Patient clinicopathological features are presented in Table 1. Ten non-tumor brain tissue samples from patients with traumatic brain injuries who underwent internal decompression served as the control group. Glioma samples were classified as grade I, II, III, or IV based on the World Health Organization (WHO) guidelines. This study was approved by the Ethical Committee of First Affiliated Hospital of China Medical University. Written informed consent was obtained from participants.

### Tissue microarray and immunohistochemistry (IHC)

All tissue specimens were fixed in 4% paraformaldehyde, embedded in paraffin, then dehydrated in an alcohol gradient and xylene until they were transparent. Paraffin-embedded tissue samples were used to construct a tissue microarray using the Manual Tissue Microarrayer (Quick-ray, UniTMA, Seoul, South Korea). IHC was performed on tissue microarray samples using routine experimental procedures 17. The primary antibodies were rabbit anti-RIOK1 (N-terminal) polyclonal antibody (1:100; ab176005, Abcam, Cambridge, UK) and AKT1 polyclonal antibody (1:100; ab194201, Abcam). An UltraSensitive^TM^ SP (Mouse/Rabbit) IHC Kit (KIT-9710, Maixin, Shenzhen, China) was used to label tissue. Scoring criteria for IHC were described in our previous publication 17.

### Cell culture and transfection

Glioma cell lines U87, U251, U373, U118, and A172 were purchased from the Cell Bank of the Chinese Academy of Sciences (Shanghai, China). All cells were cultured in Dulbecco's modified Eagle medium (Thermo Fisher Scientific, Waltham, MA, USA) supplemented with 10% fetal bovine serum (FBS) (HyClone, Logan, UT, USA) and maintained in standard conditions (5% CO_2_ and 95% atmosphere, 37°C). The U251 and U87 cell lines were transfected with RIOK1-RNAi-lentivirus from GeneChem Company (Shanghai, China) and screened by puromycin. The shRNA control sequence was 5'-UUCUCCGAACGUGUCACGUtt-3'; the shRNA RIOK1 sequence#1 was 5'-GTCATGAGTTTCATCGGTAAA-3'; and the shRNA RIOK1 sequence#2 was 5'- GGCAAATAGAATGAGAACCAT-3'.

### Immunoblot analysis

Cells and tissues were scraped in radioimmunoprecipitation assay buffer containing 1% phenylmethylsulfonyl fluoride and phosphorylase inhibitor to obtain total protein samples. The concentration was measured by a BCA kit (P0010, Beyotime, Shanghai, China). Samples (20 μg protein) were subjected to 10% sodium dodecyl sulfate-polyacrylamide gel electrophoresis and transferred to polyvinylidene fluoride membranes. After blocking with evaporated milk, the membranes were incubated with primary antibodies: RIOK1 (N-terminal) polyclonal antibody (1:1000; ab176005, Abcam), AKT1 polyclonal antibody (1:1000; ab194201, Abcam), c-Myc polyclonal antibody (1:1000; ab32072, Abcam, Cambridge, UK), glyceraldehyde 3-phosphate dehydrogenase monoclonal antibody (1:5000; 60004-1-lg, Proteintech, Wuhan, China). The blots were then labeled with appropriate secondary antibodies and visualized by enhanced chemiluminescence.

### MTT cell viability assay

Cells were seeded in 6-well plates at a density of 3000 per well. The medium was exchanged for MTT (3-(4,5-dimethylthiazol-2-yl)-2,5-diphenyltetrazolium bromide) (Sigma-Aldrich, St. Louis, MO, U.S.) solution at 0, 24, 48, or 72 h. After 2-h incubation, the MTT solution was exchanged for dimethyl sulfoxide, and the absorbance of each well was measured at 490 nm.

### Colony formation assay

Cells were seeded in 6-well plates (500-1000 cells/well) and cultured for 13 days. Cells were fixed with 4% paraformaldehyde and stained with crystal violet (Solarbio Science & Technology Co., Ltd. Beijing, China). Plates were imaged, and colonies were counted and statistically analyzed.

### Transwell assay

Cell invasion and migration abilities were evaluated with Transwell assays. Briefly, 1-3×10^4^ cells/200 µl (FBS-free medium) were seeded into the upper Transwell chamber (Corning, Corning, NY, USA), and 10% FBS medium was added to the lower chamber. After 24 h of culture, the bottom membrane of the chamber was fixed with 4% paraformaldehyde and stained with crystal violet. The cells were counted and imaged with an optical microscope (D-35578, Leica, Wetzlar, Germany). For invasion assays, the upper chambers were coated with Matrigel (BD Biosciences, Franklin Lakes, New Jersey, USA).

### Statistical analysis

All the results were analyzed by SPSS software (IBM SPSS Statistics 21, IBM Corp., Armonk, NY, USA). Correlations between RIOK1/AKT1 expression and clinicopathological characteristics were analyzed by Pearson χ^2^ test. The overall survival (OS) curves were plotted with the Kaplan-Meier method and analyzed by log-rank tests. Three or more groups were compared by one-way analyses of variance. Differential RIOK1/AKT1 mRNA expression levels were analyzed with Mann-Whitney U tests. Spearman correlation analysis was performed to assess the relationship between RIOK1 and AKT1.

## Results

### RIOK1 and AKT1 overexpression in glioma patient tissues

**Figure 1:**
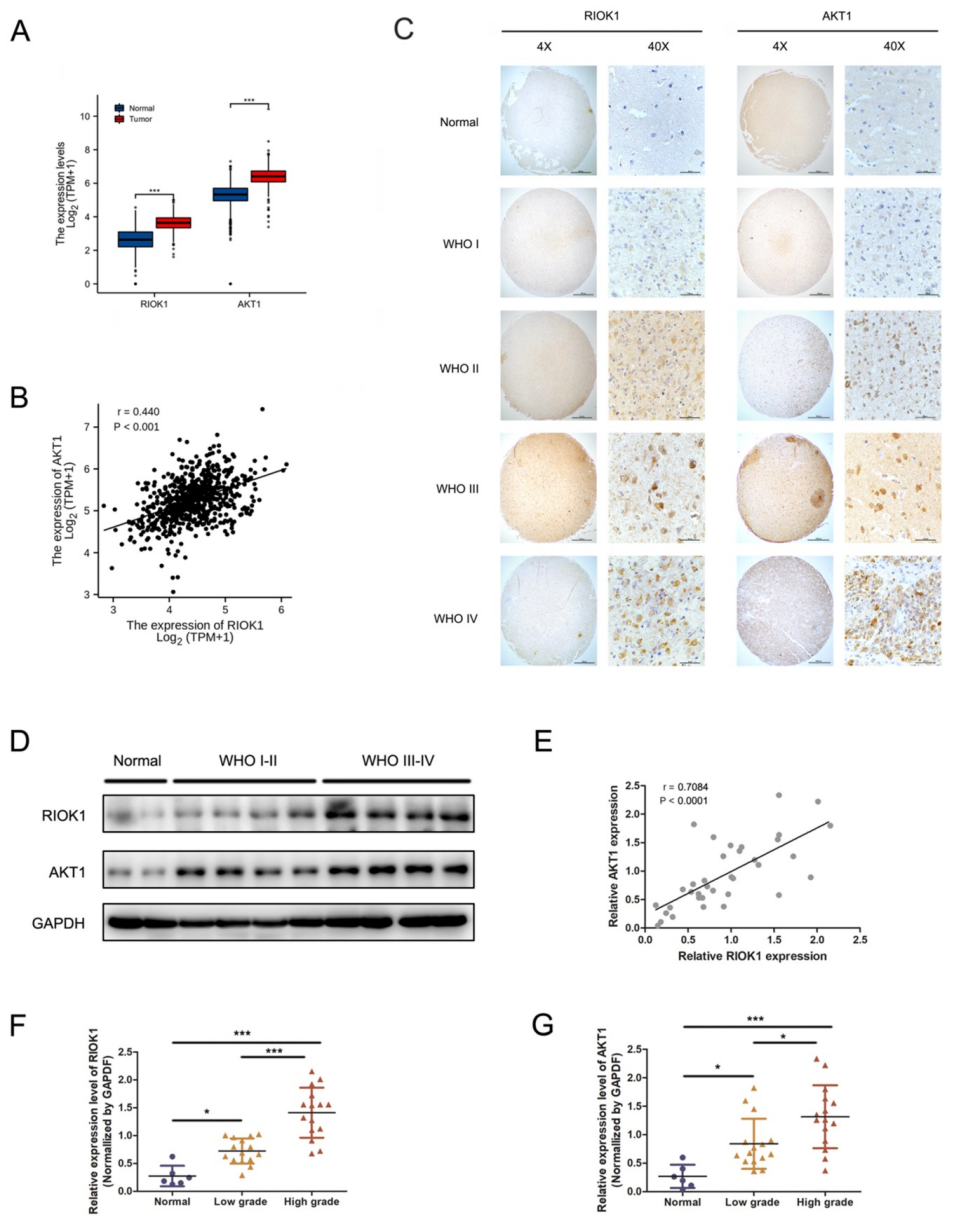
** The expression of RIOK1 and AKT1 were up-regulated in glioma tissues and correlated with the prognosis of glioma patients.** A. Data from TCGA and GTEx (RNA-seq; Normal, n=1152; glioma, n=689) showed that RIOK1/AKT1 were increased in glioma tissues compared with normal brain tissues (*** *p*<0.001). B. Correlation between RIOK1 and AKT1 expression in TCGA glioma (r=0.440, *p*<0.001). C. Relative expression of RIOK1 and AKT1 in glioma and normal tissues through IHC. D. RIOK1 and AKT1 expression in Western blot. E. Correlation between RIOK1 and AKT1 expression (r=0.7084, *p*<0.001). F, G. Relative protein expression of RIOK1 and AKT1 in 6 normal tissues and 30 glioma tissues (*p<0.05, ***p<0,001).

RIOK1 and AKT1 mRNA levels were significantly higher in glioma patient tissues compared to normal brain tissues (TCGA-GBMLGG and GTEx databases, Figure 1A), and their expression levels were significantly correlated (TCGA-GBMLGG databases, *p*<0.001, r=0.440, Figure 1B). RIOK1 and AKT1 protein levels were determined by IHC in 106 glioma and 10 control samples using tissue microarrays. RIOK and AKT1 staining intensity were weak in normal tissues but increased in glioma samples in a WHO grade-dependent fashion (Figure 1C). We performed confirmatory western blots using 6 control samples and 15 low-grade (WHO I-WHO II) and 15 high-grade (WHO III-WHO IV) glioma tissues. The results showed that RIOK1 and AKT1 protein levels were upregulated in glioma, with the highest expression in high-grade samples (Figure 1D-G and Supplementary Figure 1). Correlation analysis of the western blot data showed that RIOK1 and AKT1 expression were significantly correlated in glioma samples (*p*<0.001, r=0.7084, Figure 1E).

### Association of RIOK1 and AKT1 expression with clinicopathological characteristics and survival in glioma patients

**Figure 2:**
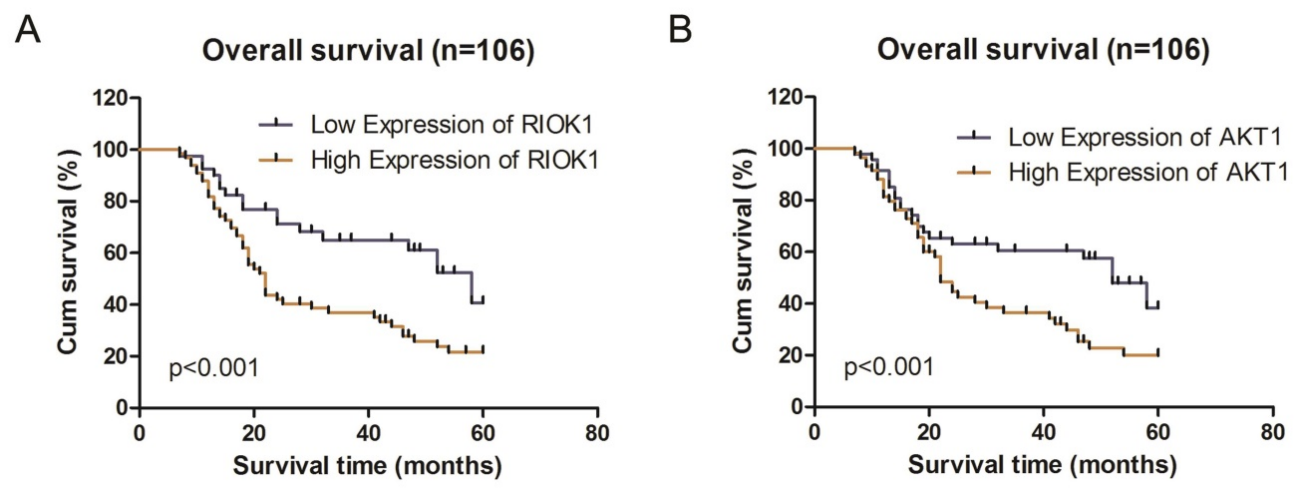
** Kaplan-Meier survival analysis according to RIOK1 and AKT1 protein expression in patients with glioma.** A. Overall survival between the high RIOK1 group and Low RIOK1 group. B. Overall survival between the high AKT1 group and Low AKT1 group.

We analyzed the associations between clinicopathological characteristics and of RIOK1 and AKT1 expression in 106 patients with GBM. RIOK1 expression was significantly associated with WHO grade (*p*=0.008) and OS (*p*=0.004). There were no significant differences for other variables including age, sex, tumor size, or Karnofsky performance status scores (*p*>0.05, Table 1). AKT1 expression was related to tumor size (*p*=0.007), WHO grade (*p*=0.022) and OS (*p*=0.034). Spearman's correlation analyses showed that the low co-expression rate of RIOK1 and AKT1 was 70.21% (33/106) and the high co-expression rate of RIOK1 and AKT1 was 88.14% (52/106, *p*<0.001) (Table 2). These results confirm RIOK1 and AKT1 expression are positively correlated in GBM tissue. The OS rate suggests that patients with high RIOK1 and AKT1 expression are more likely to have a poor prognosis (Figure 2).

### RIOK1 expression in glioma cell lines and RIOK1 stable silent cell line construction

**Figure 3:**
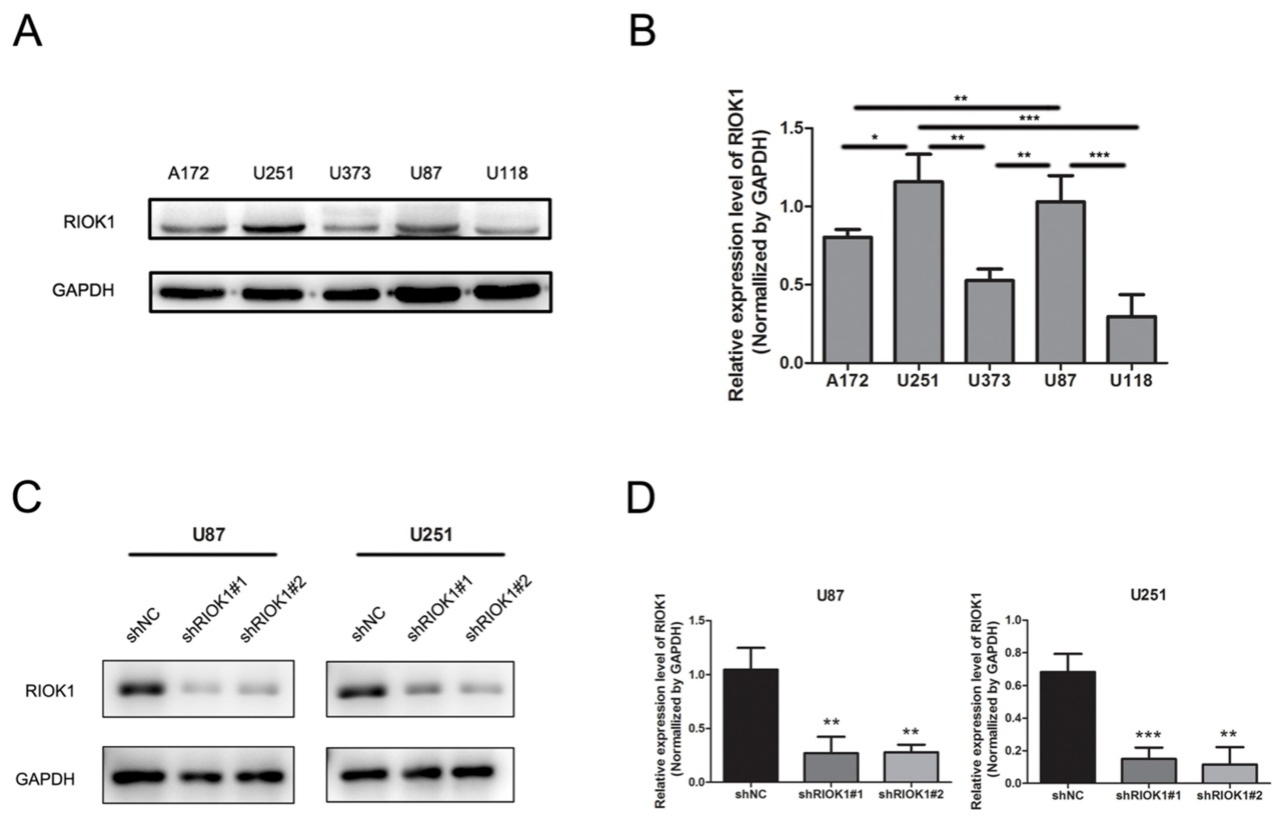
** RIOK1 expression in glioma cells and silencing RIOK1 in U87 and U251 cells.** A. The protein expression of RIOK1 in human cell lines (A172, U251, U373, U87 and U118). B. Gray value analysis of RIOK1 expression in glioma cell lines, *p<0.05, **p<0.01, ***p<0.01. C. The transfected efficiency of shRIOK1#1 and shRIOK1#2 was detected by Western blot. D. The statistical diagram of RIOK1 protein expression in each shRNA groups, **p<0.01, ***p<0.01.

RIOK1 protein expression in tumor cell lines was analyzed by western blot, which showed higher levels in U251 and U87 cell lines compared to A172, U373, and U118 cell lines (Figure 3A and B). Therefore, we silenced RIOK1 expression in U87 and U251 cell lines to clarify the function of RIOK1 in glioma cells. To avoid off-target effects, we selected two shRNAs (shRIOK1#1, shRIOK1#2) for transfection, and efficiency was detected by Western blot (Figure 3C and D).

### RIOK1 knockdown inhibits glioma cell proliferation, migration, and invasion through AKT1 and c-Myc

**Figure 4:**
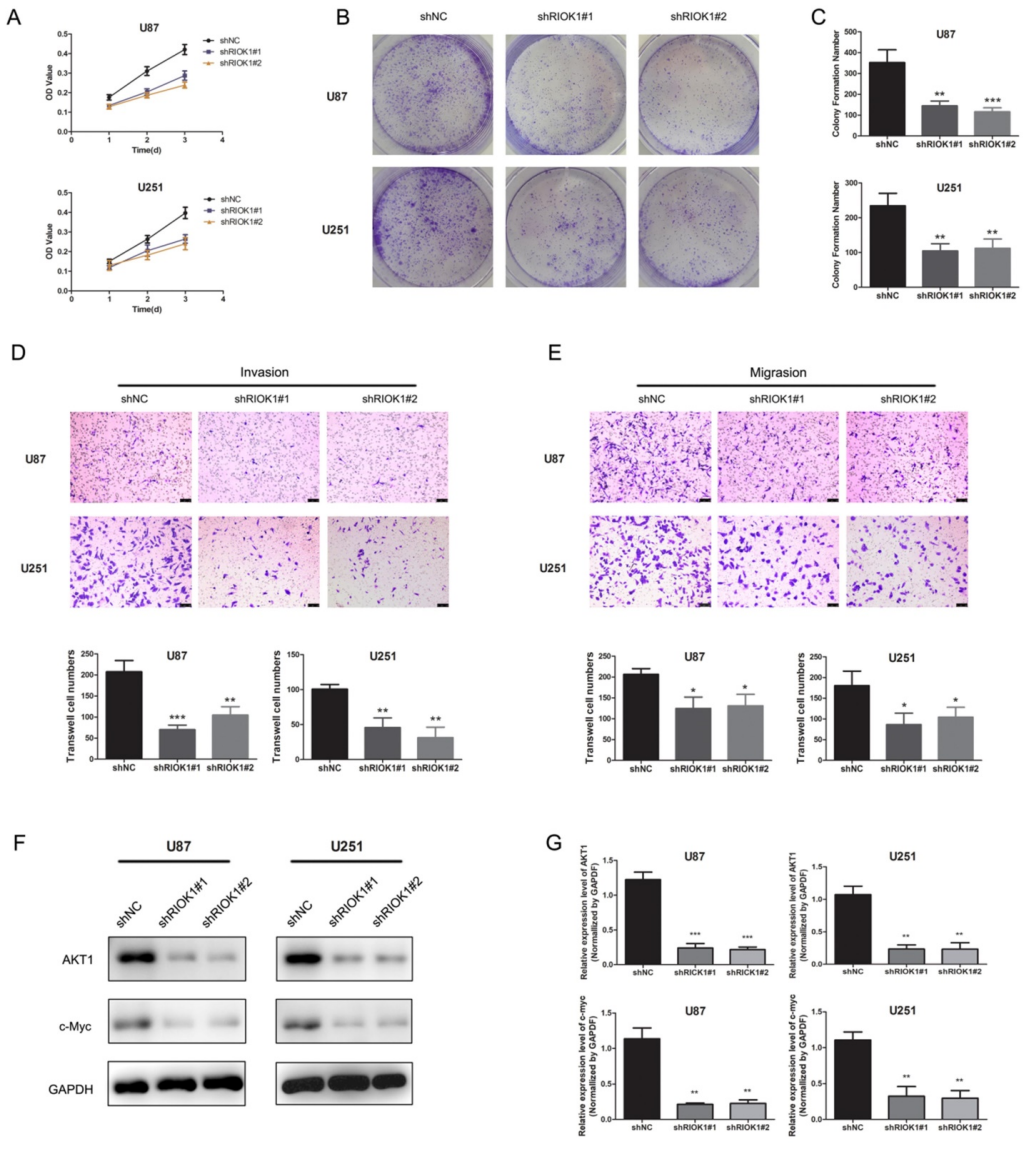
** Silencing RIOK1inhibits glioma cell growth.** A. MTT growth curve was used to evaluate cell proliferation ability after transfected with shNC, shRIOK1#1 and shRIOK1#2. B. The results of colony formation assay for U251 and U87 cells. C. The statistical diagram of colony formation assays. **p<0.01, ***p<0.001. D. Transwell invasion assays to evaluate the invasion ability of U87 and U251 cells, the corresponding statistical results are shown below the images.**p<0.01, ***p<0.001. E. Transwell migration assays to assess the migration ability of U87 and U251 cells. F, The protein expression of AKT1 and c-Myc detected by western blot. G. The statistical analysis of western blot gray value results. **p<0.01, ***p<0.001.

To evaluate the roles of RIOK1 in glioma cells, we first assessed the effect of silencing RIOK1 on glioma cell proliferation (U87 and U251). According to the MTT growth curves (Figure 4A) and colony formation experiments (Figure 4B and C), silencing RIOK1 significantly inhibited glioma cell proliferation. Transwell migration and invasion assays further showed that knockdown significantly inhibited glioma cell migration and invasion abilities (Figure 4D and E). With regard to the molecular mechanism, western blot data showed that AKT1 and c-Myc protein levels were significantly downregulated following RIOK1 knockdown (Figure 4F and G).

## Discussion

Glioma is derived from neural stromal cells including glial cells, ependymal cells, choroid plexus epithelial cells and neural parenchymal cells and is the most common primary tumor in central nervous system 18. It has high incidence, high recurrence, and high mortality rates; the 5-year OS rate is only 13%, and the median survival time is 14 months 19. Our results demonstrate that RIOK1 and AKT1 expression are upregulated in glioma and correlate with poor prognosis.

A member of the atypical kinase Rio family 20, RIOK1 is highly expressed in a variety of tumors and is involved in their malignant processes 21. Although RIOK1 has been implicated in glioma cell proliferation and invasion, its expression, clinical significance and prognostic role in glioma tissue are still unknown. AKT1 is a crucial component of AKT signaling that contributes to the proliferation, migration, and invasion of multiple tumor types including glioma 22-27. Increased AKT activity promotes glioma malignancy and cellular viability 28, 29. We investigated the co-expression of RIOK1 and AKT in GBM tissues and assessed its clinical significance. The tissue microarray results showed that RIOK1 and AKT1 expression in glioma tissues were significantly higher than those in matched normal brain tissues. In addition, tumors with higher WHO staging had the highest levels of RIOK1 and AKT1, and their expression was positively correlated. Finally, we found that both RIOK1 and AKT1 expression were correlated with poor prognosis.

Tumor cell proliferation and invasion are the main reasons leading to poor prognosis 30-33, so it is particularly important to explore possible molecular mechanisms. The human protein kinase RIOK1 is closely related to cell proliferation, and silencing RIOK1 causes cell cycle arrest 34. Researchers also found that RIOK1 can interact with the Ras protein, which often contains mutations that promote tumor growth and metastasis 35. *In vitro* studies have shown that the RIOK1-AKT signaling plays an important role in the malignant phenotype of glioma cells. Consistent with the previous studies, RIOK1 knockdown inhibited glioma cell proliferation, colony formation, migration, and invasion.

While investigating the molecular mechanism, we found that AKT1 and c-Myc expression were downregulated after silencing RIOK1. As a typical oncogenic gene, c-Myc participates in the regulation of pericellular adhesion 36, and its overexpression is closely related to the occurrence and metastasis of many kinds of tumors 37, 38. Since it is a transcription factor, c-Myc needs to enter the nucleus to regulate cell proliferation, apoptosis, and the cell cycle 39, 40. Indeed, after RIOK1 knockout, c-Myc protein levels were significantly decreased, which inhibits glioma progress. These results indicate that RIOK1 can promote glioma cell progression by acting on c-Myc and AKT1.

In summary, our results provide a theoretical basis for the importance of the RIOK1-AKT1 axis in glioma occurrence and malignant progression. The RIOK1-AKT1 pathway has the potential to become a new target for the clinical diagnosis and treatment of gliomas.

## Supplementary Material

Supplementary figure.

## Figures and Tables

**Table 1:** Clinicopathological characteristics and RIOK1/AKT1 expression in 106 glioma patients

Variables	Number of cases	RIOK1 Expression		*p*-value	AKT1 Expression		*p*-value
		High	Low		High	Low	
**Ages (years)**							
<50	37	24	13	0.686	23	14	0.324
≥50	69	42	27		36	33	
**Gender**							
Male	49	34	15	0.161	30	19	0.285
Female	57	32	25		29	28	
**Tumor size (cm)**							
<3	35	18	17	0.106	13	22	0.007**
≥3	71	48	23		46	25	
**KPS**							
<80	62	40	22	0.570	35	27	0.846
≥80	44	26	18		24	20	
**WHO**							
I	3	2	1	0.008**	1	2	0.022*
II	31	12	19		10	21	
III	26	17	9		17	9	
IV	46	35	11		31	15	
**Survival state**							
Alive	40	18	22	0.004**	17	23	0.034*
Death	66	48	18		42	24	

KPS: Karnofsky Performance Status WHO: World Health Organization *p<0.05, **p<0.001

**Table 2:** Correlation between expression of RIOK1 and AKT1 in glioma patients

	Tissues	Expression of RIOK1			
		Low (%)	High (%)	x^2-value^	*p*-value
**Expression of AKT1**	**Low Expression (%)**	33 (70.21)	14 (29.79)	37.906	<0.001
	**High Expression (%)**	7 (11.86)	52 (88.14)		

## Abbreviations

AKT1: protein kinase B
FBS: fetal bovine serum
GBM: glioblastoma multiforme
LGG: Low grade glioma
IHC: immunohistochemistry
mTOR: mammalian target of rapamycin
OS: overall survival
RIOK1: right open reading frame kinase 1
TCGA: The Cancer Genome Atlas
GTEx: Genotype-Tissue Expression
WHO: World Health Organization
