# Supplementary figures and images for: Co-overexpression of RIOK1 and AKT1 as a prognostic risk factor in glioma
Source: J Cancer. 2021 Jul 25;12(19):5745–52. doi: 10.7150/jca.60596 (PMC8408104; doi:10.7150/jca.60596)

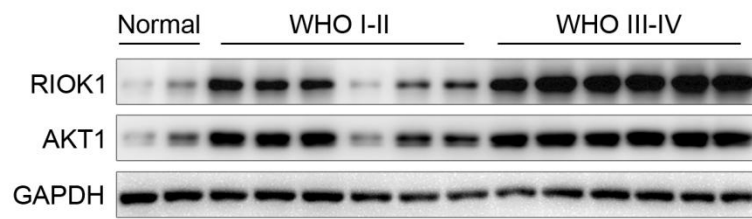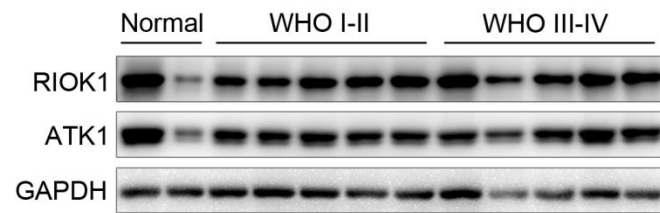

Supplementary Figure 1  
R1OK1 and AKT1 expression in Western blot.

Supplement: Supplementary file 1 — Supplementary figure. [file jcav12p5745s1.pdf]
